# Do oral contraceptives modulate the effects of stress induction on one-session exposure efficacy and generalization in women?

**DOI:** 10.1007/s00213-023-06345-3

**Published:** 2023-03-10

**Authors:** Friederike Raeder, Christian J. Merz, Martin Tegenthoff, Ekrem Dere, Oliver T. Wolf, Jürgen Margraf, Silvia Schneider, Armin Zlomuzica

**Affiliations:** 1 Faculty of Psychology, Mental Health Research and Treatment, Center, Ruhr University Bochum, Massenbergstr. 9-13, 44787 Bochum, Germany; 2 Faculty of Psychology, Institute of Cognitive Neuroscience, Department of Cognitive Psychology, Ruhr University Bochum, Bochum, Germany; 3 Ruhr University Bochum, Department of Neurology, BG-Kliniken Bergmannsheil, Bochum, Germany; 4 Institut de Biologie Paris-Seine, UFR Des Sciences de La Vie, Sorbonne Université, Paris, France

**Keywords:** Anxiety disorders, Spider fear, Stress, Glucocorticoids, Cortisol, Oral contraceptives, Single-session exposure

## Abstract

**Rationale:**

The administration of glucocorticoids (GC) as an adjunct to exposure represents a promising strategy to improve one-session exposure outcome in anxiety disorders. It remains to be determined whether similar effects can be induced with the use of acute stress. Furthermore, the possible modulation of exposure effects by hormonal factors (e.g., use of oral contraceptives (OCs)) was not explored so far.

**Objectives:**

We investigated whether acute stress prior to one-session exposure for spider fear affects its efficacy in women using oral contraceptives (*OC*) relative to free-cycling (*FC*) women. In addition, effects of stress on generalization of exposure therapy effects towards untreated stimuli were examined.

**Methods:**

Women with fears of spiders and cockroaches were randomly assigned to a *Stress* (n = 24) or *No-Stress* (n = 24) condition prior to one-session exposure. Of these 48 participants, 19 women used OC (n = 9 in the Stress, and n = 10 in the No-Stress group). All *FC* women had a regular menstrual cycle and were tested only in the follicular phase of their menstrual cycle. Pre-exposure stress induction was realized with the socially evaluated cold-pressor test. Exposure-induced changes towards treated and untreated fear stimuli were tested with behavioral approach tests for spiders and cockroaches and subjective fear and self-report measures.

**Results:**

Acute stress did not influence exposure-induced reduction in fear and avoidance of the treated stimuli (spiders). Similarly, stress had no effect on the generalization of exposure-therapy effects towards untreated stimuli (cockroaches). Exposure-induced reduction in subjective fear and self-report measures for treated stimuli was less evident in women using OC specifically after pre-exposure stress. Women using OC had higher levels of subjective fear and scored higher in self-report measures at post-treatment (24 h after exposure) and follow-up (4 weeks after exposure).

**Conclusions:**

OC intake may represent an important confounding factor in augmentation studies using stress or GC.

## Introduction

Specific fears are highly prevalent in the general population (cf. Wardenaar et al. 2017). Prevalence (Stinson et al. 2007) and incidence rates (Angst et al. 2016) are higher in females as compared to males (Kessler et al. 2005; McLean et al. 2011; Regier et al. 1990). Individuals with specific fears (e.g. fear of spiders) often also display fears to different stimuli from the same category (e.g. cockroaches) (Davey 1991; Matchett and Davey 1991). Therefore, generalization of fear responses to stimuli from the same or other categories seems to be a common phenomenon (Preusser et al. 2017; Waters et al. 2021).

Exposure therapy is an effective therapeutic intervention to reduce fear and avoidance in patients with specific fears (Hoffman and Smits 2008). The beneficial effects of exposure might be restricted to the specific stimulus used during the exposure therapy, which is suggestive of a reduced generalization of exposure-based fear reduction across different fear stimuli (Rowe and Craske 1998a, 1998b). Systematic research on the generalization of exposure across different fear stimuli has been initiated only recently (Zlomuzica et al. 2020; Preusser et al. 2017).

Behavioral interventions and pharmacological manipulations have been proposed to promote extinction learning and exposure therapy outcome (Craske et al. 2022). The administration of glucocorticoids (GCs) can potentiate the efficacy of exposure therapy, both in terms of the extent of symptom relief and shortening of the duration of therapeutic interventions (De Quervain et al. 2017, 2011; Soravia et al. 2006, 2014). The facilitating effects of glucocorticoids, however, critically depend on the frequency and timing of glucocorticoid administration (Raeder et al. 2019b; De Quervain et al. 2019).

Stress might equally promote exposure therapy outcome (Meir Drexler et al. 2018, 2019) albeit this has not been investigated so far. It is well known that acute stress activates the hypothalamus-pituitary-adrenocortical axis. Corticotropin releasing hormone secretion from the hypothalamus stimulates the pituitary gland to release adrenocorticotropic hormone (ACTH) into the blood circulation. In a final step, ACTH stimulates the adrenal glands to release glucocorticoids such as cortisol (Herman et al. 2016).

Pre-extinction stress can promote subsequent fear extinction learning and extinction retrieval (Meir Drexler et al. 2017), probably also in a sex-dependent manner (Bentz et al. 2013; Meir Drexler et al. 2019; Merz and Wolf 2017; Merz et al. 2018). Oral contraceptive (OC) use and naturally occurring changes in estrogens and progesterone can influence fear extinction (Graham and Milad 2013; Merz et al. 2018; Glover et al. 2015; Maeng and Milad 2015; Stockhorst and Antov 2016). Reduced estradiol levels in women using OCs (*OC*) relative to estradiol levels in free-cycling (*FC*) women in the early follicular phase of the menstrual cycle may account for impaired fear extinction processes (Graham and Milad 2013; Merz et al. 2018). The influence of sex, OC use and variations in estrogen levels during the menstrual cycle in the context of exposure treatment, however, has been largely neglected (but see Graham et al. 2018; Raeder et al. 2019a; Zlomuzica et al. 2020). There is some evidence for a reduced symptom relief following exposure therapy in women using OCs (Graham et al. 2018; Raeder et al. 2019a). Thus, although stress-induced elevations of cortisol concentrations prior to exposure might be suitable to promote exposure therapy efficacy, such effects on exposure therapy might be further modulated by OC intake. In addition, possible changes in stress-response in women using OC need to be considered (Jentsch et al. 2022; Kirschbaum and Hellhammer 1994; Kirschbaum et al. 1996).

In the present study, we recruited women with multiple fears (spiders and cockroaches) and asked whether pre-exposure stress induction via socially evaluated cold-pressor test (SECPT; Schwabe et al. 2008) would facilitate the exposure therapy effects. We further asked whether the beneficial effects of spider exposure would generalize to other fear stimuli (cockroaches; adapted from Preusser et al. 2017). Additionally, we investigated whether OC use in women (showing reduced estradiol levels due to OC intake), in contrast to *FC* women in the early follicular phase of the menstrual cycle (showing low estradiol levels; see Graham and Milad 2013) would influence the effect of stress on exposure efficacy and generalization. Salivary cortisol levels in healthy controls usually begin to rise in the night and have a peak in the morning after awakening, then decline over the course of the day. Exposure therapy in the morning, when cortisol levels are high, seems to be more effective in posttreatment and follow-up assessments, as compared to exposure in the evening (Lass-Hennemann and Michael 2014). To account for such time of day effect in the present study, stress was applied in the afternoon when cortisol levels are low.

## Methods

### Participants

The study was preregistered on ClinicalTrials.gov (Identifier: NCT03505437). Female participants with a fear of spiders and cockroaches were recruited via various advertisements (e.g., social media groups and online bulletin boards). Participants with a history of a mental disorder, other than a specific fear for spiders and cockroaches, were not invited to the study. Other exclusion criteria involved the presence of neurological and/or endocrine disorders, pregnancy, current pharmacological treatment, and current smoking (> than five cigarettes per month). Finally, participants with a body mass index (BMI) greater than 27 or smaller than 19 kg/m^2^ and shift-workers were excluded.

A total of 104 individuals took part in a pre-experimental telephone screening to check for inclusion/exclusion criteria. Participants were not required to fulfill the diagnosis of a specific phobia although they had to present a substantial degree of fear towards spiders and/or cockroaches. The latter was ascertained on the basis of specific self-report measures, i.e. the Fear of spiders questionnaire (FSQ; Rinck et al. 2002) and the Fear of cockroaches questionnaire (FCQ; Scandola et al. 2010). After this screening, 77 individuals remained eligible for participation. A total of 29 individuals dropped out of the study for one of the following reasons: No longer interested in participation (n = 13), unable to attend (n = 4) or not showing up for their appointment (n = 12).

The final sample, therefore, included 48 women who were assigned to either the stress or the No-Stress group. In particular, 15 *FC* and 9 *OC* women were included to the No-Stress groups, while 14 *FC* and 10 *OC* women were included in the Stress group. All *FC* women had a regular menstrual cycle and were tested only in the follicular phase of their menstrual cycle (i.e., between the 3^rd^ and 9^th^ day after menstruation onset). *FC* and OC status as well as the exact phase of the menstrual cycle were determined via self-reports using a standardized questionnaire. *OC* women were tested during their OC intake phase. Participants were instructed to abstain from eating, drinking, or smoking and not to engage in any kind of excessive exercise for approximately 90 min prior to each of the three experimental sessions. The exclusion criteria and abstinence instructions have been selected in accordance with the pertinent literature in order to reduce confounding factors that have been shown to affect acute stress-induced salivary cortisol concentrations (De Punder et al. 2019; Kirschbaum et al. 1993, 1995, 1996; Kudielka et al. 2004, 2009).

All experimental procedures were approved by the ethics committee of the Faculty of Psychology of the Ruhr University Bochum and carried out in accordance with the Declaration of Helsinki. All participants provided written informed consent and received a compensation of 60€ for the completion of the study.

### Measures

#### Anxiety and depression questionnaires

Possible differences in anxiety and depression levels between the stress and No-Stress group were assessed with the Beck depression inventory-II (BDI-II; Hautzinger et al. 2006) and the State-trait anxiety inventory (STAI-S and STAI-T; Laux 1981). The BDI-II is a 21-item questionnaire used for assessing self-rated severity of depression in both clinical and nonclinical samples. The STAI consists of 40 items for measuring two types of anxiety, i.e. state anxiety and trait anxiety.

#### Spider and cockroach fear-related questionnaires

Different aspects related to the fear of spiders and cockroaches were assessed by using a battery of questionnaires. We used (1) the German version of the FSQ (Rinck et al. 2002), which includes 18 items that are scored on a 7-point Likert scale, (2) the Spider phobia questionnaire (SPQ; Hamm 2006) which comprises 31 dichotomously coded (i.e., true vs. false) items, and (3) the Spider beliefs questionnaire (SBQ; Pössel and Hautzinger 2003) which measures dysfunctional beliefs on 48 items according to a scale from 0 (“not at all”) to 100 (“completely”). For fear of cockroaches, the FCQ (Scandola et al. 2010), which is an exact adaptation of the FSQ but only relating to cockroaches, and a modified version of the SBQ to reflect beliefs about cockroaches (see Botella et al. 2010) were applied. Higher scores reflect a higher level of fear in each of the questionnaires.

#### Behavioral approach test (BAT)

**Fig. 1:**
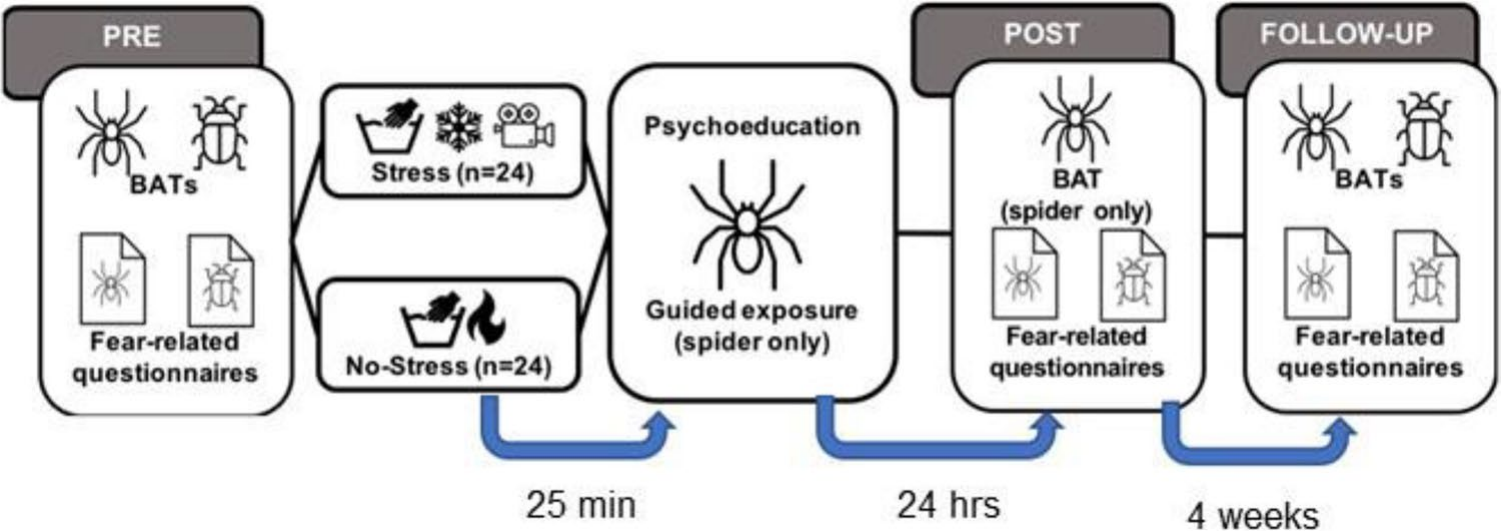
Outline of the experimental design and main outcome measures. At the beginning of the experiment the participants underwent two BATs (spider and cockroach), and thereafter, presented with spider and cockroach-related questionnaires (Pre-SECPT treatment stage). Thereafter, the participants underwent the SECPT either under the stress condition with hands submerged in cold water or under the no-stress condition with hands submerged in warm water. Twenty-five minutes after the SECPT, all participants received a short psychoeducation and in-vivo guided spider exposure. After a delay of 24 h, the spider BAT was repeated and both the spider and the cockroach fear-related questionnaires were presented (Post-treatment phase). Long-term effects of exposure were evaluated four weeks later (Follow-up phase). At follow-up, both spider and cockroach BATs were repeated and spider and the cockroach fear-related questionnaires were presented. Abbreviations: BAT: Behavioral avoidance test, SECPT: Socially evaluated cold-pressor test

Both fear and avoidance of spiders and cockroaches were assessed with separate and independent BATs at the beginning of the first experimental session (“Pre”), at the post-stress-treatment and guided spider exposure efficacy evaluation stage (“Post”), as well as during the long term fear reduction stability assessment and generalization stage (“Follow-up”; Fig. 1; adapted from Preusser et al. 2017). To this end, the fearful stimulus (spiders: Tegenaria domestica, 1 cm; cockroaches: Blaptica dubia, 4 cm) was placed in a transparent plastic container at the far end of a 3 m x 3 m room. Participants were instructed to perform different steps with increasing difficulty. The steps involved entering the room, approaching the fearful stimulus as fast as possible, removing it from the jar of container one and ultimately placing it into the jar of container two, which was located at a distance of 1.5 m to container one. On each step, participants were instructed to only continue their approach behavior if they experienced that the fear is tolerable. When the participants indicated that fear is intolerable for continuation, the BAT was stopped.

Both the spider and cockroach BATs were scaled in approach distance which comprised fifteen different steps according to a modified and expanded version of the BAT introduced by Lass-Hennemann and Michael (2014). The steps ranged from 0 (= refusal to enter the test room) to steps 1–13 (entering the room and approaching the terrarium placed at the corner of the room, touching the terrarium, placing the hand inside the terrarium etc.) and the final step 14 (= successfully placing the stimulus into a target jar that was positioned in another container).

#### Subjective units of distress scale (SUDS)

The participant’s subjective fear at the initial and final approach distance during the BAT was recorded (Raeder et al. 2019a, b). The SUDS with scores ranging from 0 (= no fear) to 100 (= excessive fear) served as the primary measure of subjective fear (reported verbally) during the BAT and the exposure session.

#### Socially evaluated cold-pressor test (SECPT)

Participants were exposed to either the stress or the control condition of the SECPT (adapted from Schwabe et al. 2008). Participants in the Stress group were instructed to place their dominant hand, including their forearm, into a small tub which contained ice water with a temperature between 0 °C and 3 °C for a duration of three minutes. An additional experimenter was present, observing and videotaping the participant during the procedure. If participants were not able to tolerate the cold water for the duration of three minutes, they were instructed to place their arm above the water in the repository. Participants in the control group had to place their dominant hand into warm water with a temperature of 36 °C to 37 °C. To assess activation of the sympathetic nervous system, blood pressure (i.e., systolic and diastolic (mmHg)) was assessed at nine times in total. These referred to three times before (baseline), during (peak), and after (post) the stress induction or no-stress control procedure. Mean values were computed for each assessment time. After the procedure, participants had to indicate on a scale from 0 (i.e., “not at all”) to 100 (i.e., “very much”) how difficult, unpleasant, stressful, and painful the experience was felt (ratings adapted from Schwabe et al. 2008).

#### Salivary cortisol

Salivary cortisol concentrations were measured as a marker for HPA axis activity (Kirschbaum and Hellhammer 1994). Samples were taken with saliva sampling tubes (Sarstedt, Nümbrecht, Germany) on different time points across the course of the experiment (please refer to Table 1 for a precise overview). On Day 1, which included the pre-treatment screening, SECPT and exposure session, samples 1–7 were taken. On Day 2, which included the post-treatment BAT for spiders another two samples (8–9) were taken. On Day 3, which included the follow-up assessment with BATs for spiders and cockroaches, another two samples (10–11) were taken. Chemiluminescence immunoassay with high sensitivity (IBL International, Hamburg, Germany) was used to measure salivary cortisol concentrations. The intra- and inter-assay coefficients of variance for cortisol were both below 8.0%.

**Table 1:** Times of salivary cortisol measurements

Sample	Experimental Phase	Salivary Control Sample Times
1	Day 1, Phase 1	Start of pre-treatment assessment
2	Day 1, Phase 1	25 min after BAT onset
3	Day 1, Phase 1	Arrival at the stress laboratory
4	Day 1, Phase 1	After questionnaire completion / before stress induction
5	Day 1, Phase 1	25 min after stress induction
6	Day 1, Phase 1	25 min after start of the exposure session
7	Day 1, Phase 1	End of the exposure session
8	Day 2, Phase 2	Start of post-treatment assessment
9	Day 2, Phase 2	25 min after BAT onset
10	Day 3, Phase 3	Start of follow-up assessment
11	Day 2, Phase 3	25 min after BAT onset

*Note.* The most relevant cortisol measures in this paper were sample number 4 and sample number 5. These correspond to Baseline (Sample 4) and Post-stress induction (Sample 5)

### One-session exposure treatment

The exposure session consisted of the first eight steps of a standardized fourteen-step fear hierarchy progression procedure (steps increased in difficulty; see Mystkowski et al. 2006 for details). The experimenter assessed individual levels of fear (SUDs) by questioning the participant during the performance of each step. Each step needed to be performed until the participant reached a SUDs rating of 30 or lower. The exposure session was terminated after 45 min or once the participant had completed all steps.

### Experimental design and procedure

The study comprised three experimental phases which were conducted on three separate days (see Fig. 1), all of which were conducted in the afternoon (1 to 5 p.m.) to reduce daytime-related variability in stress hormone secretion. The first phase on Day 1 started with two BATs (first spider BAT, then cockroach BAT) and the completion of the BDI-II, STAI, as well as the spider and cockroach-fear related questionnaires (i.e., SPQ, FSQ/FCQ, SBQ/CBQ). After that, participants had to complete either the cold water (Stress group) or the warm water (No-Stress group) condition of the SECPT. Thereafter, participants received a brief psychoeducation session about specific phobia and subsequently engaged in the in-vivo guided spider exposure, which commenced 25 min after the participants had immersed their hand into the water. The entire first phase on Day 1 lasted approximately 140 min. The second phase on Day 2 (of approximately 45 min duration) took place 24 h later after the first phase and involved a post-treatment measurement consisting of spider BAT and both the spider and the cockroach fear-related questionnaires. The third phase (of approximately 45 min duration) took place four weeks later and involved a follow-up assessment. Here, both BATs as well as the completion of the spider and the cockroach fear-related questionnaires were conducted. At the end of the third phase, participants were debriefed and received their compensation.

### Statistical analyses

Statistical analyses were performed with SPSS Statistics for Macintosh, Version 27.0 (Armonk, NY: IBM Corp.). Pre-exposure participant characteristics and stress ratings were compared using a series of two-way ANOVAs with the between-subjects factors “*Stress*” (*Stress* vs. *No-Stress*) and “*OC*” (*OC* vs. *FC*). Blood pressure as well as salivary cortisol were analyzed with ANOVAs with the within-subjects factor “*Time*” (salivary cortisol: baseline vs. 25 min after SECPT; blood pressure: measurements at baseline, during stress induction vs. after the SECPT) and the between-subjects factors “*Stress*” and “*OC*”*.* Exposure-induced changes in outcome measures (i.e., BAT score, subjective fear during the BAT, questionnaires) were also analyzed with two-way ANOVAs with three levels of the within-subjects factor “*Time*” (Day 1 *Pre, Day 2 Post,* vs. Day 3 *Follow-up*). Note that analyses of the cockroach BAT score and subjective fear at the initial and final approach distances comprised only two levels (*Pre* vs. *Follow-up*). ANOVA results were considered significant when P-values smaller than 0.05 were found. Bonferroni-corrected multiple pair-wise group comparisons were made with T-tests for independent samples and considered to be significant when p-values smaller than P = 0.016 emerged.

## Results

### Pre-exposure participant characteristics

As displayed in Table 2, all experimental groups were comparable with respect to age (Main effect of *OC*: F(1,44) = 1.659; P = 0.204; Main effect of *Stress*: F(1,44) = 0.280; P = 0.599; Interaction: F(1,44) = 0.491; P = 0.487; two-way ANOVA; Table 2), BMI (Main effect of *OC*: F(1,44) = 1.129; P = 0.294; Main effect of *Stress*: F(1,44) = 0.0001; P = 0.991; Interaction: F(1,44) = 0.735; P = 0.396), and their scores on the BDI-II (Main effect of *OC*: F(1,43) = 0.771; P = 0.385; Main effect of *Stress*: F(1,43) = 1.369; P = 0.248; Interaction: F(1,43) = 0.981; P = 0.327), and the STAI-T before exposure (Main effect of *OC*: F(1,44) = 0.262; P = 0.611; Main effect of *Stress*: F(1,44) = 0.323; P = 0.573; Interaction: F(1,44) = 1.987; P = 0.166). We additionally compared *FC* and *OC* groups with respect to demographical data, salivary cortisol, blood pressure and stress ratings separately for No-Stress and Stress conditions. No significant differences between *FC* and *OC* groups were found for these comparisons (all Ps > 0.1; see Table 3 for a listing of comparisons and corresponding P-values).

**Table 2:** Overview of participant characteristics, salivary cortisol, blood pressure and stress ratings

	No-Stress						Stress					
	*FC* women (N = 14)		*OC* women (N = 9)		**Total (N = 23)**		*FC* women (N = 13)		*OC* women (N = 10)		**Total (N = 23)**	
	M	SD	M	SD	M	SD	M	SD	M	SD	M	SD
*Demographics*												
Age (years)	22.73	3.54	24.67	3.08	**23.46**	**3.44**	23.93	2.84	24.50	3.66	**24.17**	**3.14**
BMI (kg/m^2^)	22.03	2.70	21.87	2.10	**21.97**	**2.45**	22.71	3.20	21.21	2.01	**22.09**	**2.81**
STAI-T	40.07	7.54	37.33	8.11	**39.04**	**7.70**	41.00	11.52	40.90	10.09	**40.96**	**10.72**
STAI-S	46.80	10.04	43.89	7.54	**45.71**	**9.12**	44.07	14.32	50.30	9.23	**46.67**	**12.61**
BDI	9.40	7.08	5.56	5.79	**7.96**	**6.77**	9.77	8.25	10.00	5.44	**9.87**	**7.02**
*Salivary cortisol (nmol/l)*												
Baseline	3.85	2.58	2.92	1.39	**3.48**	**2.21**	3.08	2.15	2.76	1.33	**2.94**	**1.81**
25 min after stress	2.80	1.85	2.30	1.10	**2.60**	**1.59**	4.39	3.00	4.48	5.10	4.43*	**3.94**
*Blood pressure*												
Systolic (mmHg)												
Baseline	104.53	8.43	110.63	12.28	**106.82**	**10.24**	106.38	8.24	111.17	10.74	**108.38**	**9.45**
Peak	105.64	9.40	108.37	10.16	**106.67**	**9.56**	125.05	15.47	129.87	19.41	127.06**	**16.99**
Post	103.16	9.70	107.15	9.71	**104.65**	**9.69**	109.60	9.22	112.23	12.30	**110.69**	**10.44**
Diastolic (mmHg)												
Baseline	59.84	5.77	67.44	9.37	**62.69**	**8.06**	63.57	7.71	66.83	7.43	**64.85**	**7.60**
Peak	65.18	8.10	69.56	8.35	**66.82**	**8.30**	79.81	12.76	83.44	11.11	81.23**	**12.02**
Post	62.20	7.53	66.78	8.01	**63.92**	**7.87**	66.69	7.85	67.89	11.25	**67.16**	**9.10**
*Stress ratings*												
Difficulty	1.33	3.52	2.50	7.07	**1.74**	**4.91**	78.57	21.43	82.00	20.44	80.00**	**20.64**
Unpleasant	7.33	18.70	1.25	3.54	**5.22**	**15.34**	82.86	20.16	79.00	29.61	81.25**	**24.01**
Stressful	2.00	7.75	2.50	7.07	**2.17**	**7.36**	67.14	26.73	70.00	24.94	68.33**	**25.48**
Painful	0.00	0.00	0.00	0.00	**0.00**	**0.00**	85.00	22.10	82.00	23.94	83.75**	**22.42**

^*^ *p* < .05 ** *p* < .001 compared to the no-stress group

**Table 3:** Comparison of main demographic characteristics, salivary cortisol, blood pressure and stress ratings between the *OC* and *FC* group in the No-Stress and Stress conditions

	*FC* vs. *OC* (No-Stress)	*FC* vs. *OC* (Stress)
	P-value; t-test for independent samples	
*Demographics*		
Age (years)	0.1883	0.6706
Marital status	0.5273	0.6303
Education	0.5044	0.8965
BMI (kg/m^2^)	0.8800	0.2055
STAI-T	0.4118	0.9826
STAI-S	0.4613	0.2411
BDI	0.1835	0.9400
*Salivary cortisol (nmol/l)*		
Baseline	0.3370	0.6860
25 min after stress	0.4807	0.9563
*Blood pressure*		
Systolic (mmHg)		
Baseline	0.1625	0.2290
Peak	0.5112	0.5053
Post	0.3400	0.5535
Diastolic (mmHg)		
Baseline	**0.0216**	0.3265
Peak	0.2183	0.4918
Post	0.1730	0.7658
*Stress ratings*		
Difficulty	0.6000	0.6976
Unpleasant	0.3772	0.7071
Stressful	0.8809	0.7933
Painful	N.c	0.7544

*Note:* The most relevant cortisol measures in this paper were sample number 4 and sample number 5. These correspond to Baseline (Sample 4) and Post-stress induction (Sample 5). For corresponding group means and standard deviations see Table 2. Significant p-values are indicated by bold numbers. N.c. = Not calculated; only zero values in both groups

Most importantly, there were no pre-treatment group differences in the spider- and cockroach-fear related questionnaires (FSQ: Main effect of *OC*: F(1,44) = 1.228; P = 0.274; Main effect of *Stress*: F(1,44) = 2.331; P = 0.134; Interaction: F(1,44) = 1.023; P = 0.317, FCQ: Main effect of *OC*: F(1,44) = 1.770; P = 0.190; Main effect of *Stress*: F(1,44) = 0.298; P = 0.588; Interaction: F(1,44) = 0.470; P = 0.496), as well as in subjective fear at the initial approach distance to the spider and the cockroach during the pre-treatment BAT (Spider SUDS: Main effect of *OC*: F(1,44) = 0.210; P = 0.886; Main effect of *Stress*: F(1,44) = 0.446; P = 0.508; Interaction: F(1,44) = 0.309; P = 0.581, Cockroach SUDS: Main effect of *OC*: F(1,44) = 1.067; P = 0.307; Main effect of *Stress*: F(1,44) = 0.187; P = 0.668; Interaction: F(1,44) = 0.054; P = 0.817).

The analysis of approach behavior during the pre-treatment BAT yielded a significant main effect of *OC* (Spider approach: Main effect of *OC*: F(1,44) = 12.010; P = 0.001, Cockroach approach: Main effect of *OC*: F(1,44) = 12.758; P = 0.001). Women who used OC showed less approach behavior during the pre-treatment BAT with the spider (M = 4.79, SD = 1.40) and the cockroach (M = 5.00, SD = 1.92) as compared to the *FC* group (Spider: M = 6.69, SD = 2.04; Cockroach: M = 7.17, SD = 2.07). In contrast, no significant main effect of the grouping factor *Stress* (Spider approach: Main effect of Stress: F(1,44) = 0.406; P = 0.527, Cockroach Approach Index: Main effect of *Stress*: F(1,44) = 0.026; P = 0.873) or a *OC* x *Stress* interaction effect was found for approach behavior during the pre-treatment BAT measurement (Spider approach: Interaction: F(1,44) = 0.084; P = 0.773, Cockroach Approach Index: Interaction: F(1,44) = 0.026; P = 0.873).

### Stress-response manipulation check

#### Stress-effects on salivary cortisol concentrations

**Fig. 2:**
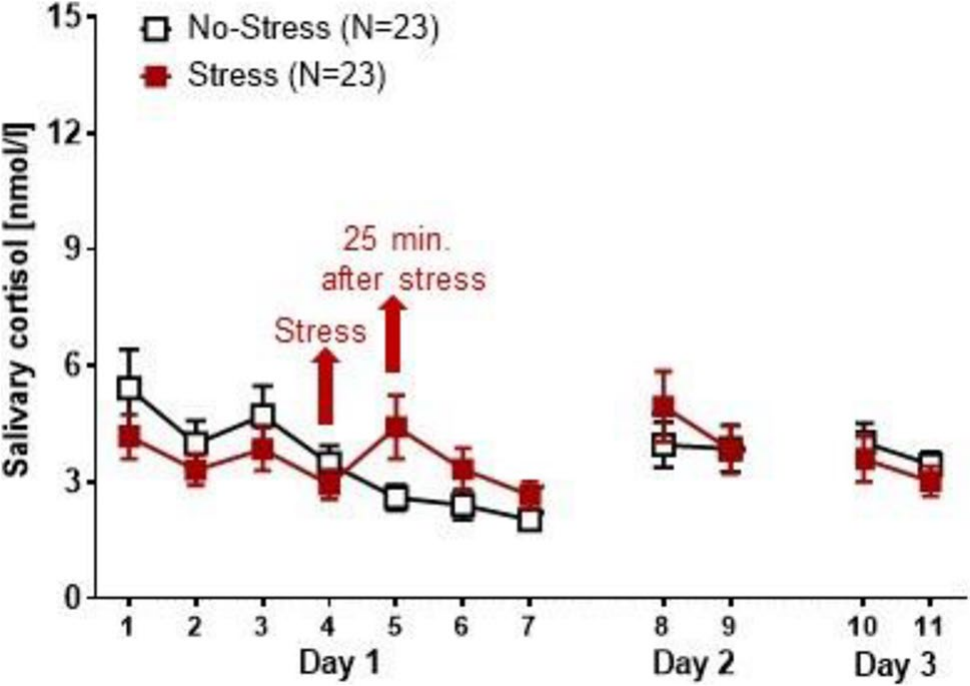
Monitoring of salivary cortisol concentrations of the Stress and No-Stress groups. Each data point represents mean and SEM salivary cortisol concentrations that have been measured during 7 sampling points on experimental day 1, two sampling points on day 2 and another two sampling points on experimental day 3 (see Table 1 for details)

Two participants were excluded from data analysis, because of undetectable cortisol concentrations, resulting in a sample size of 23 participants in both the Stress and No-Stress groups. The trajectory of salivary cortisol concentrations across the measures 1–7 on experimental Day 1, measures 8–9 on experimental Day 2, and measures 10–11 on Day 3 is illustrated in Fig. 2. In order to determine whether the stress induction would raise salivary cortisol concentrations, we compared the last measurement before stress induction (sample point 4 on experimental day one) with the first measurement after stress induction (sample point 5) using a two-way ANOVA with repeated measures. As expected, stress induction significantly increased salivary cortisol concentrations as indicated by a significant interaction of the factors *Time* and *Stress* (F(1,42) = 7.573; P = 0.009; Table 2). Main effects of the factors *Time*, *OC* and *Stress* were not statistically significant (*Time*: F(1,42) = 0.636; P = 0.430, *OC*: F(1,42) = 0.395; P = 0.533, *Stress*: F(1,42) = 1.183; P = 0.283). The remaining first and second order interactions between the factors *Time*, *OC* and *Stress* were also all non-significant (*OC* vs. *Stress*: F(1,42) = 0.209; P = 0.650, *Time* x *OC*: F(1,42) = 0.245; P = 0.623, *Time* x *OC* x *Stress*: F(1,42) = 0.0000146; P = 0.990). Post-hoc group comparisons indicated that the Stress and No-Stress groups had similar cortisol concentrations at baseline (T[44] = 0.912; P = 0.367; T-test for independent samples), but 25 min after the stress induction, participants exposed to cold water (Stress group), showed significantly higher salivary cortisol concentrations as compared to the warm-water exposed participants (No-Stress group: T[28.995] = -2.059; P = 0.049; Fig. 2).

#### Stress effects on systolic and diastolic blood pressure

Assessments of systolic and diastolic blood pressure (cf. Table 2) at baseline, during stress induction and post-stress stages also indicated a successful stress induction. Two-way ANOVA with repeated measures yielded significant effects of the factors *Time* and *Stress* as well as significant *Time* x *Stress* interactions (Systolic blood pressure: Main effect of *Time*, F(2,88) = 38.824; P < 0.001; Main effect of *Stress*, F(1,44) = 8.799, P = 0.005; *Time* x *Stress* interaction: F(2,88) = 35.381; P < 0.001, Diastolic blood pressure: Main effect of *Time*, F(2,86) = 54.002; P < 0.001; Main effect of *Stress*, F(1,43) = 6.903, P = 0.012; *Time* x *Stress* interaction: F(2,86) = 22.353; P < 0.001). The effect of stress induction on systolic and diastolic blood pressure was not affected by the *OC* factor. No significant main effect of the factor *OC*, *Time* x *OC* interaction, or *OC* x *Stress* interaction was found (Systolic blood pressure: Main effect of *OC*, F(1,44) = 1.839, P = 0.182; *Time* x *OC* interaction: F(2,88) = 0.437; P = 0.647; *OC* x *Stress* interaction: F(1,44) = 0.001; P = 0.976, Diastolic blood pressure: Main effect of *OC*, F(1,43) = 3.025, P = 0.089; *Time* x *OC* interaction: F(2,86) = 0.741; P = 0.480; *OC* x *Stress* *interaction: F(1,43) = 0.356; P = 0.554).*

Post-hoc analyses indicated that Stress and No-Stress groups showed similar systolic and diastolic blood pressure during the baseline measurement prior to stress induction (Systolic blood pressure: T[46] = -0.547; P = 0.587, Diastolic blood pressure: T[45] = -0.941; P = 0.352). As expected the Stress group had significantly higher systolic and diastolic blood pressure during the stress induction as compared to the No-Stress group (Systolic blood pressure: T[46] = -5.124; P < 0.001, Diastolic blood pressure: T[45] = -4.802; P < 0.001). The Stress group showed a significantly higher systolic, but not diastolic, blood pressure after the SECPT as compared to the No-Stress group (Systolic blood pressure: T[46] = -2.077; P = 0.043, Diastolic blood pressure: T[45] = -1.308; P = 0.197), suggesting that there was a small after-effect of stress induction, that, however, as indicated by the diastolic blood pressure measure, was likely in a process of rapid decline. In line with the significant effects of the stress-treatment on physiological parameters, the Stress group also experienced the SECPT as being more difficult (Main effect of *Stress*: F(1,43) = 283.708; P < 0.001; Table 2), unpleasant (Main effect of *Stress*: F(1,43) = 153.232; P < 0.001), stressful (Main effect of *Stress*: F(1,43) = 129.612; P < 0.001), and painful (Main effect of *Stress*: F(1,43) = 286.950; P < 0.001), as compared to the subjective stress ratings of the No-Stress group.

#### Effects of stress and OC on spider exposure efficacy: Spider BAT Behavioral approach/avoidance

The participants’ approach behavior towards spiders increased across the 3 BAT trials (Main effect of *Time*: F(2,88) = 97.334; P < 0.001; data not shown). The *OC* group showed less approach behavior as compared to the *FC* group as indicated by a main effect of the factor *OC* (F(1,44) = 13.768; P < 0.001). In contrast, approach behavior was not modulated by the factor *Stress* (Main effect of *Stress*: (F(1,44) = 0.244; P = 0.624). Furthermore, no significant *OC* x *Stress* interaction was found (F(1,44) = 0.244; P = 0.624). First and second order interactions that included the factor *Time* were all non-significant (*Time* x *OC*: F(2,88) = 0.260; P = 0.772, *Time* x *Stress*: F(2,88) = 0.202; P = 0.817, *Time* x *OC* x *Stress*: F(2,88) = 0.864; P = 0.425).

### Subjective fear ratings (SUDS)

The analysis of the initial approach distance measure of the BAT suggested that the participants experienced a reduction in their subjective fear levels across the 3 trials (Main effect of *Time*: F(2,88) = 79.625; P < 0.001). The subjective fear reduction was modulated by both the factors *OC* and *Stress* as indicated by a significant second order interaction between *Time, OC* and *Stress* (F(2,88) = 3.135; P = 0.048). First order interactions between *Time* and *Stress* (F(2,88) = 0.820; P = 0.922; Two-way ANOVA with repeated measures), *Time* and *OC* (F(2,88) = 1.427; P = 0.246) and *OC* and *Stress* (F(2,88) = 1.357; P = 0.250), were all statistically not significant. Furthermore, the main effects of the factors *OC* and *Stress* were also not significant (*OC*: F(1,44) = 1.908; P = 0.174, *Stress*: F(1,44) = 0.328; P = 0.570).

**Fig. 3:**
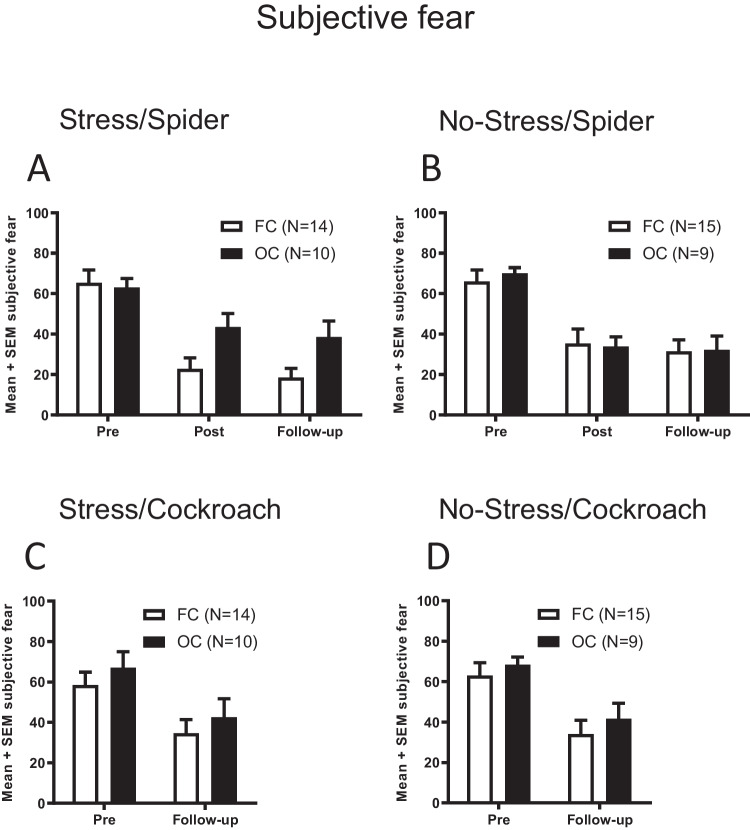
Subjective fear at the initial approach distance. A-B. Subjective fear ratings (SUDS) during the spider-BAT during pre-exposure, post-exposure, and follow-up measurements of the Stress and No-Stress groups. **C.-D.** Subjective fear ratings during the cockroach-BAT during pre-exposure, and follow-up measurements of the Stress and No-Stress groups. Bars represent mean and SEM of the SUDS scores. Abbreviations: FC: Free-cycling, OC: Oral contraceptive, SUDS: Subjective units of distress Scale

Post-hoc analyses of the Stress group indicated that *OC* and *FC* groups experienced similar levels of subjective fear during the pre-stress session (T[21.489] = -0.303; P = 0.764; T-test for independent samples; Fig. 3A). Trends for higher levels of subjective fear in *OC* group were found during post-stress (T[22] = -2.420; P = 0.024), and follow-up sessions (T[22] = -2.344; P = 0.028). However, these differences failed to reach the level of statistical significance after Bonferroni-correction. No significant differences in subjective fear levels were observed in the No-Stress *OC* and *FC* groups (Pre: T[19.858] = -0.628; P = 0.537; Post: T[21.4747] = 0.167; P = 0.869; Follow-up: T[22] = -0.077; P = 0.939; Fig. 3B).

The subjective fear levels at the final approach distance during the pre- and follow-up BATs suggested that the participants showed a significant reduction of their subjective fear assessments (Main effect of *Time*: F(2,78) = 22.554; P < 0.001). No significant main effects of the factors *OC* and *Stress* were found (*OC*: F(1,39) = 0.000069; P = 0.993. Stress: F(1,39) = 0.305; P = 0.584). First and second order interactions were likewise statistically not significant *OC* x *Stress*: F(1,39) = 1.493; P = 0.229, *Time* x *OC*: F(2,78) = 0.474; P = 0.624, *Time* x *Stress*: F(2,78) = 1.314; P = 0.275, *Time* x *OC* x *Stress*: *F(2,78) = 1.552; P = 0.218).*

### Spider fear-related questionnaires

#### Fear of spider questionnaire (FSQ)

**Fig. 4:**
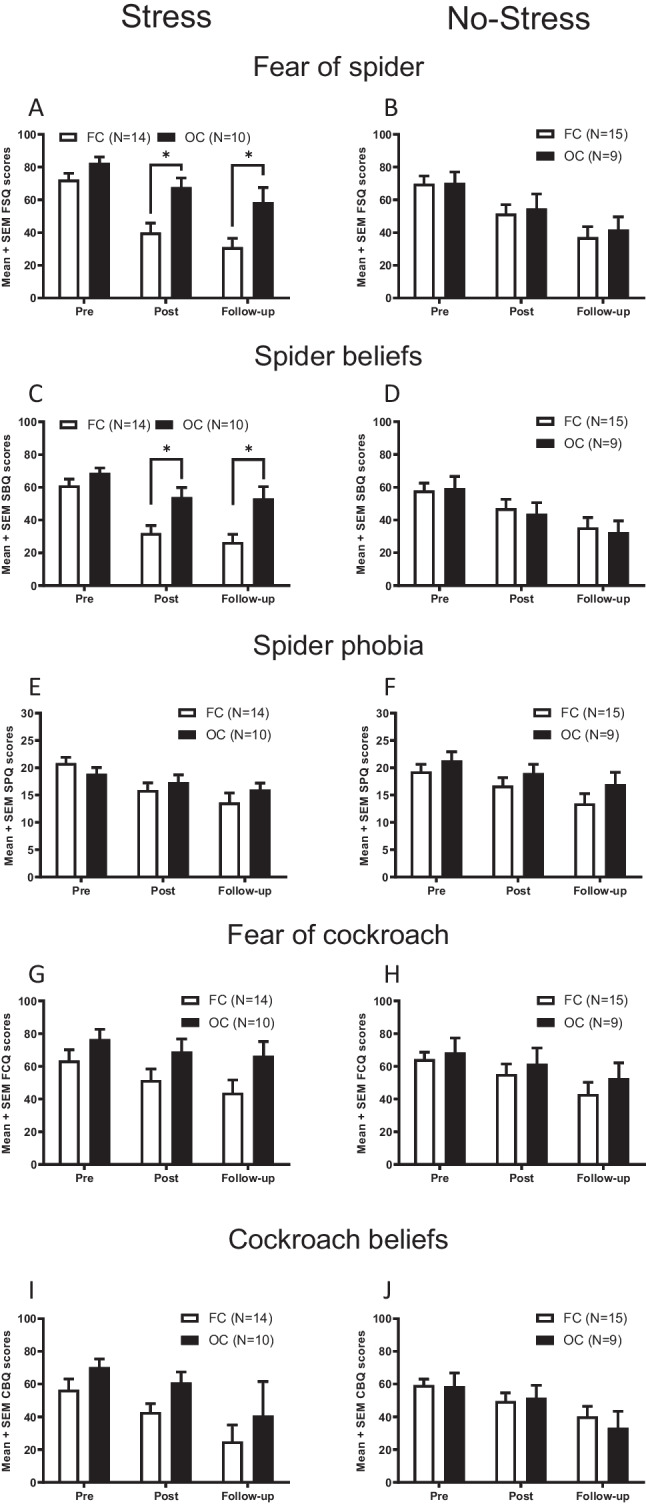
Spider and cockroach-related questionnaires. Pre-, post-exposure and follow-up assessment of spider- and cockroach-related questionnaires of the Stress and No-Stress groups. A.-F. Bars represent mean and SEM of the fear of spiders, spider beliefs and spider phobia scores, respectively. G.-J. Bars represent mean and SEM of the fear of cockroach, and cockroach beliefs scores, respectively. Abbreviations: FC: Free-cycling, OC: Oral contraceptive, FSQ: Fear of spiders questionnaire, SBQ: Spider beliefs questionnaire, SPQ: Spider phobia questionnaire, FCQ: Fear of cockroaches questionnaire, CBQ: Cockroach beliefs questionnaire. *: p < 0.05; T-test for independent samples

Across the 3 measurements (*Pre, Post,* and *Follow-up*), participants showed a significant decrease in their fear of spider scores as indicated by a significant main effect of *Time* (F(2,88) = 60.783; P < 0.001; Figs. 4A,B). Furthermore, a significant mean effect of the factor *OC* was found, suggesting that the *OC* group expressed higher levels of spider fear as compared to the *FC* group (F(1,44) = 5.550; P = 0.023). Post-hoc analyses indicated that this effect was mainly driven by significant differences between *OC* and *FC* groups in the Stress condition. This difference was observed after the induction of stress and during the follow-up measurement, but not prior to the stress induction (*Pre*: T[22] = -1.834; P = 0.080; *Post:* T[22] = -3.374; P = 0.003; *Follow-up:* T[22] = -2.758; P = 0.011). No such difference between the *OC* and *FC* group was observed in the No-Stress condition (*Pre*: T[22] = -0.059; P = 0.954; *Post:* T[22] = -0.325; P = 0.748; *Follow-up:* T[22] = -0.453; P = 0.655). In contrast, no significant main effect of the factor *Stress* was found (F(1,44) = 0.726; P = 0.399). First and second order interactions between the factors *Time*, *OC* and *Stress* were all non-significant (*Time* x *OC*: F(2,88) = 2.178; P = 0.119, *Time* x *Stress*: F(2,88) = 0.677; P = 0.511, *OC* x *Stress*: F(1,44) = 3.331; P = 0.075, *Time* x *OC* x *Stress*: F(2,88) = 0.986; P = 0.377).

#### Spider beliefs questionnaire (SBQ)

Participants showed a significant decrease in their spider belief scores across the Pre, Post and Follow-up measurements (Main effect of *Time*: F(2,88) = 64.356; P < 0.001; Figs. 4C,D). No significant main effects of the factors *OC* or *Stress* were observed (*OC:* F(1,44) = 2.963; P = 0.092, *Stress:* F(1,44) = 0.416; P = 0.522). However, significant interactions were observed between the factors *OC* and *Stress* (F(1,44) = 4.157; P = 0.047), as well as between the factors *Time* x *OC* x *Stress* (F(2,88) = 3.829; P = 0.025). Similar to the fear of spiders results reported above, post-hoc analyses indicated that this effect was mainly driven by significant differences between *OC* and *FC* groups in the *Stress* condition. *OC* and *FC* groups showed a significant difference in their spider belief scores after the induction of stress and during the follow-up measurement, but not prior to the stress induction (*Pre:* T[22] = -1.494; P = 0.149; *Post:* T[22] = -2.920; P = 0.008; *Follow-up*: T[22] = -3.234; P = 0.004). Again no such difference between the *OC* and *FC* groups was observed in the *No-Stress* condition (Pre: T[22] = -0.198; P = 0.845; Post: T[22] = 0.398; P = 0.695; Follow-up: T[22] = 0.303; P = 0.765). Interactions between the factors *Time* and *OC* (F(2,88) = 1.315; P = 0.273) and *Time* and *Stress* (F(2,88) = 2.386; P = 0.098) were not statistically significant.

#### Spider phobia questionnaire (SPQ)

Similar to the fear of spider and spider beliefs results presented above, participants showed a significant decrease in their spider phobia scores across the *Pre, Post*, and *Follow-up* measurements (Main effect of *Time*: F(2,88) = 37.888; P < 0.001; Figs. 4E,F). Furthermore, a significant *Time* x *OC* interaction was found (F(2,88) = 3.197; P = 0.046), which was obviously mainly driven by the relatively stable spider phobia scores of the *OC* groups. No significant main effects of the factors *OC* or *Stress* were obtained (*OC:* F(1,44) = 1.310; P = 0.259, *Stress*: F(1,44) = 0.240; P = 0.627). Similarly, no significant interaction effects were observed (*OC* x *Stress*: F(1,44) = 0.492; P = 0.487, *Time* x *OC*: F(2,88) = -0.289; P = 0.750, *Time* x *Stress*: F(2,88) = 1.089; P = 0.341).

In sum, these results suggest *OC* use in combination with stress appears to reduce some of the benefits of exposure therapy on some self-report measures.

### Effects of OC and Stress on the generalization of guided spider exposure effects to other fear stimuli: Cockroach BAT

#### Behavioral approach/avoidance

The participants’ approach behavior towards cockroaches increased from the pre-exposure measurement to the follow-up BAT measurement (Main effect of *Time*: F(1,44) = 54.189; P < 0.001; data not shown). The *OC* group showed less approach behavior as compared to the *FC* group, as evidenced by a main effect of the factor *OC* (F(1,44) = 10.595; P = 0.002).

Approach behavior towards cockroaches was not affected by the factor *Stress* (Main effect of *Stress*: (F(1,44) = 0.023; P = 0.881). First and second order interactions were all non-significant (*OC* x *Stress*: F(1,44) = 0.066; P = 0.798, *Time* x *OC*: F(1,44) = 0.124; P = 0.727, *Time* x *Stress*: F(1,44) = 0.000074; P = 0.993, *Time* x *OC* x *Stres*s: F(1,44) = 0.488; P = 0.488). Post-hoc pairwise comparisons of the pre-exposure approach behavior of the *OC* and *FC* groups indicated that the *OC* groups generally showed higher avoidance of the cockroaches as compared to the *FC* groups (*Stress* condition: T[22] = 2.269; P = 0.033, *No-Stress* condition: T[22] = 2.838; P = 0.010). Most remarkably, the spider exposure intervention abolished this difference irrespective of the stress condition (*Stress* condition: T[22] = 1.765; P = 0.091, *No-Stress* condition: T[22] = 1.484; P = 0.152). These results suggest that the effects of spider exposure intervention indeed generalized to a different insect fear stimulus and consequently reduced avoidance behavior.

#### Subjective fear ratings (SUDS)

The subjective fear levels at the initial approach distance during the pre- and follow-up BATs suggested that participants experienced a significant reduction in their subjective fear levels (Main effect of *Time*: F(1,42) = 50.104; P < 0.001; Two-way ANOVA with repeated measures; Fig. 3C,D). No significant main effects of the factors *OC* and *Stress* were found (*OC*: F(1,42) = 1.341; P = 0.253. *Stress*: F(1,42) = 0.005; P = 0.943). First and second order interactions were likewise statistically not significant (*OC* x *Stress*: F(1,42) = 0.001; P = 0.974, *Time* x *OC*: F(1,42) = 0.016; P = 0.900, *Time* x *Stress*: F(1,42) = 0.090; P = 0.765, *Time* x *OC* x *Stress*: F(1,42) = 0.000039; P = 0.995).

The subjective fear levels at the final approach distance measured during the *Pre*- and *Follow-up* BATs did not show a significant decline in experienced fear level (Main effect of *Time*: F(1,43) = 3.249; P = 0.078). No significant main effects of the factors *OC* and *Stress* were found (*OC*: F(1,43) = 0.245; P = 0.623. *Stress*: F(1,43) = 0.327; P = 0.570). First and second order interactions were likewise statistically not significant (*OC* x *Stress*: F(1,43) = 0.057; P = 0.812, *Time* x *OC*: F(1,43) = 0.460; P = 0.501, *Time* x *Stress*: F(2,78) = 0.002; P = 0.968, *Time* x *OC* x *Stress*: F(1,43) = 0.288; P = 0.594).

### Cockroach fear-related Questionnaires

#### Fear of cockroach questionnaire (FCQ)

Participants showed a significant decrease in their fear of cockroach scores across the 3 assessments (*Pre, Post*, and *Follow-up*) as indicated by a significant main effect of *Time* (F(2,88) = 21.525; P < 0.001; Figs. 4G,H). No significant main effects of the grouping factors *OC* or *Stress* was found (*OC*: F(1,44) = 3.111; P = 0.085, *Stress*: F(1,44) = 0.376; P = 0.543). First and second order interactions between the factors *Time, OC* and *Stress* were all non-significant (*Time* x *OC*: F(2,88) = 1.075; P = 0.346, *Time* x *Stress*: F(2,88) = 0.572; P = 0.567, *OC* x *Stress*: F(1,44) = 0.635; P = 0.430, *Time* x *OC* x *Stress*: F(2,88) = 0.083; P = 0.920).

#### Cockroach beliefs questionnaire (CBQ)

Similar to the fear of cockroach scores, participants showed a significant decrease in their cockroach belief scores across the *Pre, Post* and *Follow-up* measurements (Main effect of Time: F(2,88) = 17.437; P < 0.001; Figs. 4I,J). No significant main effects of the factors *OC* or *Stress* were observed (*OC*: F(1,44) = 1.110; P = 0.298, *Stress*: F(1,44) = 0.007; P = 0.933). First and second order interactions between the factors *Time*, *OC* and *Stress* were all non-significant (*Time* x *OC*: F(2,88) = 0.200; P = 0.819, *Time* x *Stress*: F(2,88) = 0.418; P = 0.660, *OC* x *Stress*: F(1,44) = 1.782; P = 0.189, *Time* x *OC* x *Stress*: F(2,88) = 0.125; P = 0.883).

## Discussion

We have investigated the effects of acute stress on one-session exposure outcome and generalization in women with fear of spiders and cockroaches. Acute stress, administered prior to exposure, had no overall effect on exposure-induced reduction in fear and avoidance for the treated stimuli (spiders). Likewise, participants’ approach behavior towards untreated stimuli (cockroaches) increased from the pre-stress and pre-exposure measurement to the follow-up measurement, but no effect of stress induction was found. Similar findings have been obtained for subjective and self-report measures. This pattern of results suggests no effect of stress on exposure efficacy and generalization in women. However, the exposure-induced reduction in subjective fear and self-report measures for treated stimuli was reduced in women using OC specifically after pre-exposure stress induction. These results underline the importance of sex hormones as potential modulators of the efficacy and outcome of an one-session exposure treatment.

Our finding differs from previous exposure therapy augmentation studies showing that GC administration (that is associated with high cortisol levels) prior to exposure therapy leads to stronger symptom reduction as compared to a placebo condition (De Quervain et al. 2011; Soravia et al. 2006, 2014). Furthermore, it has been reported that higher physiological morning cortisol levels prior to exposure lead to stronger symptom alleviation as compared to lower evening cortisol levels prior to exposure (Lass-Hennemann and Michael 2014).

In contrast to these studies, the present results suggest that acute stress (and the accompanying physiological cortisol increase) does not promote exposure-induced reduction of fear and avoidance towards treated stimuli in women. More specifically, in *FC* stress did not lead to additional beneficial reduction of fear and avoidance. Women using OCs also did not benefit from acute stress, but show a less pronounced reduction in subjective fear at post-treatment and long term follow-up.

The absence of beneficial effects of pre-exposure stress on exposure efficacy and generalization was not due to a failure to induce stress. A multi-factorial manipulation check demonstrated that the Stress groups showed increased blood pressure, salivary cortisol concentrations and stress ratings after stress induction as compared to a baseline measurement immediately before stress induction. More specifically, *FC* women showed a slightly increased stress cortisol response. Our results also suggest that OC use does not attenuate the cortisol response to acute stress, as it was previously shown with other stressors, e.g. the Trier Social Stress Test (Gervasio et al. 2022) or bicycle ergometry (Kirschbaum et al. 1996). Thus, the modulatory effect of OC use on a one-session exposure outcome in female participants exposed to acute stress cannot be attributed to an insufficient cortisol response. However, it should be noted that the effects of laboratory stress on cortisol responses might be further dependent on the menstrual cycle phase (both in the presence or absence of OC use, see Montero-López et al. 2018). This possible confounding factor should be addressed systematically in future studies on this topic. It is noteworthy, however, that our and previous studies cannot be directly compared due to differences in exposure session protocols and sample characteristics. It has also been shown that patients with specific fears respond differently than normal volunteers to a stressor associated with social evaluation (Furlan et al. 2001). While a proportion of social phobia patients shows an increase in salivary cortisol to a social speech task, some patients show a decrease in cortisol (Furlan et al. 2001). Such a dichotomy in magnitude and in distribution of the cortisol response to a stressor might also be present in our study. This might explain why the cortisol increase in the stress group and in *FC* women in particular is somewhat lower than expected. Unfortunately, the small sample size in our study limits the possibility to test this conclusion. The same might be true for *OC* women with specific fears being exposed to stress (see Jentsch et al. 2022) who might equally show an altered pattern of cortisol responses after stress. These factors might collectively explain why the relatively low cortisol increase after stress in our study was insufficient to replicate previous findings on the beneficial effect of GCs on exposure efficacy. Stress, at least induced by the SECPT, in contrast to GC administration (De Quervain et al. 2011; Soravia et al. 2006, 2014) or daytime dependent increases in cortisol (Las-Hennemann & Michael, 2014) might be insufficient to induce cortisol levels to a point where exposure therapy effects can be further boosted. Additionally, more potent stress protocols in increasing cortisol concentrations such as the TSST should be investigated in future studies. We and others showed that OC use diminishes the exposure-induced benefit in female participants with spider phobia (Graham et al. 2018; Raeder et al. 2019a). While OC intake had no effect on the efficacy of exposure on the reduction of spider fear and avoidance under No-Stress conditions, we found a detrimental effect of OC intake on exposure efficacy under the pre-exposure Stress condition. This finding is in line with previous reports that demonstrate an antagonistic effect of OC on cortisol-induced facilitation of emotional learning in women (Merz and Wolf 2017; Merz et al. 2012). A possible explanation of this effect might be the blockade of brain estradiol synthesis by hormonal contraceptives that is exaggerated by stress. It has been shown that women taking hormonal contraceptives (causing reduced estradiol concentrations) compared to free-cycling women in the follicular phase of the menstrual cycle (showing low estradiol levels) show impairments in fear extinction memory (Graham and Milad 2013). In a study with a mixed group of men and women (Pletzer et al. 2021), social evaluative stress modulated estradiol levels in a bidirectional way. Two subgroups of responders were identified. About sixty percent of the participants showed a reduction in estradiol levels after 20 min post-stress, while the remaining 40 percent of the participants responded with an increase in estradiol levels at this stage. Most importantly, these changes were not correlated with the cortisol response to social evaluative stress (Pletzer et al. 2021). Similarly, women with low endogenous estradiol levels show a more pronounced recovery of extinguished fear measured at the level of skin conductance responses as compared to women with high estradiol levels (White and Graham 2016). Additionally, stress exposure prior to fear acquisition modulates the recall of fear extinction memories in dependence of the estradiol status of women (Antov and Stockhorst 2014; reviewed in Hsu et al. 2021). Most importantly, women with high estradiol status seem to be insensitive to declarative memory impairments induced by pre-learning stress (Antov and Stockhorst 2018). Therefore, it is conceivable that stress and OC had an additive suppressive effect on estradiol synthesis up to a point where it affected extinction learning leading to a diminution of the exposure effect on fear and avoidance reduction. Future studies should incorporate hormonal analyses to gain more insight into these mechanisms.

Another aim of this study was to investigate possible effects of stress on generalization of exposure therapy effects towards untreated stimuli (Preusser et al. 2017; Pittig et al. 2018). Research on such stimulus-based generalization of exposure therapy effects is limited (Rowe and Craske 1998a, 1998b). We previously showed that exposure therapy to alleviate spider fear and avoidance also leads to the reduction of fear and avoidance towards cockroaches (Preusser et al. 2017). This finding indicates a generalization of exposure-induced benefit across different fear categories. In the present study, we have successfully replicated this finding. Although we did not incorporate a non-treated control group, the increase in approach behavior towards cockroaches is substantial and comparable to our previous finding (Preusser et al. 2017). This suggests that exposure towards a specific fear stimulus might be helpful for coping with other fear stimuli that belong to the same fear category, for example small animal phobia in the present study.

Therapy generalization effects might be related to a generalized mastery experience or an increase in self-efficacy beliefs after exposure to the treated stimuli (Raeder et al. 2019c). Enhanced self-efficacy can promote extinction learning and retrieval (Zlomuzica et al. 2015) which in turn enhances learning experiences made during exposure therapy (Raeder et al. 2020). Whether such generalization effects can also be observed for stimuli that belong to a different fear category (Zlomuzica et al. 2020) such as heights (relative to spiders for example), remains to be explored.

Nevertheless, stress did not influence the generalization of exposure-induced benefit across different fear stimuli. Basic research on the effects of stress on stimulus-based fear extinction is very limited (but see Hagedorn et al. 2021). Findings from basic research on context-dependent generalization suggests that stress promotes generalization of extinction memories across different contexts (Meir Drexler et al. 2017) and abolishes context-dependent fear renewal (Meir Drexler et al. 2018). Acute stress might support the generalization of therapeutic effects from the treatment context to other everyday situations (Vervliet et al. 2013). The present findings, however, suggest that this conclusion does not apply for (stimulus-based) generalization of therapeutic effects from treated to untreated fear stimuli. Whether stress supports context-dependent generalization of therapy effects but not generalization of exposure-therapy effects across different stimuli remains to be explored.

Potential limitations of our study might be related to the relatively small sample size or individual differences in the amount of cortisol released following a lab stressor (van Eck et al. 1996) and other effects (i.e. experienced life stress, wake time, BMI, and caffeine intake) in this relationship. It remains to be shown whether cortisol increases or other factor are responsible for the observed effects. In order to estimate the probability of a type 2 error, that is false negative results, we performed a post-hoc power-analysis using G*Power to assess the archived power. Post-hoc power-analyses were performed on spider-BAT subjective fear ratings at the post-exposure, and follow-up stage of the Stress groups. The post-hoc power analysis yielded an achieved power (1-β error probability) of 0.9994 and 0.99811 for post-exposure and follow-up data, which points to a lower possibility of reporting false negative results. Nevertheless, in future studies, using a larger sample, it might be interesting to investigate whether the systemic administration of GC to women using *OC* and *FC* might affect the efficacy of exposure-treatment and the generalization of symptom alleviation to related and unrelated fear stimuli such as spiders, cockroaches and heights.

## Conclusion

Our data highlight the complexity of the relationship between stress, hormonal status, one-session exposure outcome, and exposure-treatment generalization. Further research on the physiological, neurohormonal and cognitive mechanisms of exposure-treatment generalization is needed to obtain a more consistent picture which can be translated into innovative therapeutic approaches.

## Data Availability

All data obtained during the current study are available from the corresponding author on reasonable request.
